# Dynamics of cell wall assembly during early embryogenesis in the brown alga *Fucus*

**DOI:** 10.1093/jxb/erw369

**Published:** 2016-10-06

**Authors:** Thomas A. Torode, Amandine Siméon, Susan E. Marcus, Murielle Jam, Marie-Anne Le Moigne, Delphine Duffieux, J. Paul Knox, Cécile Hervé

**Affiliations:** ^1^Centre for Plant Sciences, Faculty of Biological Sciences, University of Leeds, Leeds, UK; ^2^Sorbonne Universités, UPMC Univ Paris 06, UMR 8227, Integrative Biology of Marine Models, Station Biologique de Roscoff, CS 90074, Roscoff, France; ^3^CNRS, UMR 8227, Integrative Biology of Marine Models, Station Biologique de Roscoff, CS 90074, Roscoff, France

**Keywords:** Alginate, arabinogalactan-protein, brown algae, cell wall, *Fucus serratus*, monoclonal antibody, sulfated fucan, zygote.

## Abstract

Zygotes from *Fucus* species have been used extensively to study cell polarization and rhizoid outgrowth, and in this model system cell wall deposition aligns with the establishment of polarity. Monoclonal antibodies are essential tools for the *in situ* analysis of cell wall glycans, and here we report the characteristics of six monoclonal antibodies to alginates (BAM6–BAM11). The use of these, in conjunction with monoclonal antibodies to brown algal sulfated fucans, has enabled the study of the developmental dynamics of the *Fucus* zygote cell walls. Young zygotes are spherical and all alginate epitopes are deposited uniformly following cellulose deposition. At germination, sulfated fucans are secreted in the growing rhizoid wall. The redistribution of cell wall epitopes was investigated during treatments that cause reorientation of the growth axis (change in light direction) or disrupt rhizoid development (arabinogalactan-protein-reactive Yariv reagent). Alginate modeling was drastically impaired in the latter, and both treatments cause a redistribution of highly sulfated fucan epitopes. The dynamics of cell wall glycans in this system have been visualized *in situ* for the first time, leading to an enhanced understanding of the early developmental mechanisms of *Fucus* species. These sets of monoclonal antibodies significantly extend the available molecular tools for brown algal cell wall studies.

## Highlight

New monoclonal antibodies to brown algal cell wall polymers allow tracing of *in situ* dynamics of alginates and sulfated fucans in *Fucus* zygotes and in the context of growth disruption

## Introduction

Brown algae are a large and diverse class of marine organisms ubiquitous to coastal environments. These photosynthetic eukaryotes exhibit complex multicellularity and have cells surrounded by a cell wall, two features that they share with land plants and also with green and red algae (Popper *et al.*, 2011). However, their evolutionary origin is not related to these other groups, and the brown algae have evolved both multicellularity and cell walls independently from the other multicellular photosynthetic lineages. Brown algae belong to the stramenopiles, a phylum which also contains unicellular (e.g. various protists and diatoms) and filamentous (e.g. oomycetes) members. Through the course of evolution, this lineage gained new biosynthetic capabilities for cell wall components (Michel *et al.*, 2010), allowing the evolution of cell walls/extracellular matrices that were certainly critical for the generation of complex multicellularity (Michel *et al.*, 2010; Deniaud-Bouët *et al.*, 2014).

Brown algal cell walls are composed predominantly of polysaccharides together with lower amounts of phenols, proteins, and halide compounds (Deniaud-Bouët *et al.*, 2014). Sulfated fucans and the alginates are the major sets of matrix polysaccharides, which are by far prevalent over crystalline polymers including cellulose. Sulfated fucans encompass a highly diverse spectrum of sulfated polysaccharides containing α-l-fucose residues. Heterofucans may also contain galactose, mannose, xylose, and uronic acids. Alginate is a linear co-polymer of 1,4-linked uronic acid epimers differing only at C5: β-d-mannuronic acid (M) and α-l-guluronic acid (G). These residues are arranged in homopolymeric regions of MM- and GG-blocks, interspaced with random arrangements of both monomers (MG-blocks). While the MM-block regions do not associate in the presence of divalent cations, GG-block regions will form ‘egg-box’ junctions with calcium (Grant *et al.*, 1973), bridging two antiparallel chains and leading to gel formation. The alginate polysaccharide in brown algae is first produced as a pure mannuronate structure. Subsequently this alginate is modified at the polymer level by the action of mannuronan C5-epimerases (ManC5-Es), which catalyze the conversion of M residues into G residues. This enzymatic activity allows the fine-tuning of the alginate structure, with the generation of variants having distinct chemical and rheological properties.

Additional cell wall components in brown algae are shared with land plants, including cellulose, although it is a minor fraction of brown algal walls (from 0% to 10%). A β-1,3-glucan, known as callose in land plants, is also present in brown algal walls (Raimundo *et al.*, 2015), where it can be transiently expressed upon infection (Tsirigoti *et al.*, 2014). Arabinogalactan-proteins (AGPs) are also cell surface components in brown algae (Hervé *et al.*, 2016). AGPs are a complex family of proteoglycans that have been known for decades to be present at cell surfaces in land plants. Although they are reported to play key roles in a number of cellular and developmental processes in these organisms, the detailed molecular mechanisms involved remain elusive (Siefert and Roberts, 2007).

In the Fucales order of brown algae, zygotes have long served as an important developmental biology model system to study the mechanisms by which cells acquire polarity and regulate asymmetric cell division (Bisgrove and Kropf, 2008). In the case of *Fucus* spp. zygotes, cell wall deposition has been demonstrated to be an absolute requirement for polar axis fixation (Kropf *et al.*, 1988) and cell fate selection (Berger *et al.*, 1994). Past studies, conducted to locate the main classes of polymers tentatively at the cell surface of developing *Fucus* zygotes, have suggested that cellulose and alginates are the first polysaccharides to be deposited uniformly into the wall after fertilization (Quatrano and Stevens, 1976; Quatrano, 1982). Sulfated fucans are proposed to be deposited at a later stage during polar axis establishment, and specifically at the rhizoid pole featuring apical growth (Quatrano and Stevens, 1976; Bisgrove and Kropf, 2001), where they were suggested to be involved in the formation of an axis-stabilizing complex (ASC) (Quatrano *et al.*, 1991). Recent work has demonstrated that AGPs are expressed in the early wall, along with alginates and cellulose, before being spatially restricted to the wall opposite the rhizoid pole (Hervé *et al.*, 2016). Although cell wall components have been shown to play major roles in early embryogenesis in *Fucus* species, there were few accessible molecular tools at the time to investigate selectively the detailed distribution of polysaccharide motifs. Most attempts to map cell wall polymers at the cell surface of *Fucus* zygotes used labor-intensive chemical extractions and/or colorimetric methods (Quatrano and Stevens, 1976; Quatrano *et al.*, 1985), birefringence assays (Bisgrove and Kropf, 2001), or general stains which are not specific enough to ascertain the exact nature of the targeted polymer (Quatrano and Stevens, 1976; Bisgrove and Kropf, 2001). Such methods do not readily allow the cell-based location of polymers or their interactions during wall assembly and modeling or in response to environmental cues. It could be argued that this drawback has played a part in the arrest of studies in this developmental model system in recent decades.

We recently isolated four monoclonal antibodies (MAbs) named the brown alga monoclonal (BAM) antibodies (BAM1 to BAM4) that bind to sulfated fucans and heterofucan preparations (Torode *et al.*, 2015). Here we report the generation of six MAbs (BAM6–BAM11) directed towards different block structures of alginate. We have now used these two sets of molecular probes to investigate cell wall deposition during the development of *Fucus serratus* zygotes. We have reinvestigated and extended the findings outlined above on the spatial and temporal distribution of cell wall polymers during early embryogenesis in this system. Moreover, we have studied the impact of the loss of AGP function on cell wall assembly. The results provide evidence that implicates AGPs both in alginate modeling and in the distribution of some fucan populations in the *F. serratus* zygote.

## Materials and methods

### Polysaccharides and oligosaccharides

Alginate (A7003), medium viscosity alginate (A2033), low viscosity alginate (A2158), fucoidan (F5631), laminaran (L9634), gum Arabic (G9752), oat spelt xylan, and polygalacturonic acid (P3889) were obtained from Sigma-Aldrich. A sodium alginate from *Laminaria hyperborea* stipes was kindly provided by DuPont (Landerneau, France). High viscosity alginate (02154723, MP Biochemicals) and very low viscosity alginate (A18565, Alfa Aesar) were kindly provided by Dr Richard Blackburn (University of Leeds). Tamarind xyloglucan, potato galactan, guar galactomannan, sugar beet arabinan and citrus pectin were obtained from Megazyme International (Bray, Ireland).

The MM- and GG-blocks of alginates were prepared using the DuPont sodium alginate according to Heyraud *et al.* (1996). A 0.5% alginate solution was hydrolyzed for 5 h at 100 °C in 0.3 M HCl. After selective precipitation and centrifugation, the blocks were dialyzed, freeze-dried, and re-suspended in distilled water, and respective M/G compositions monitored by NMR. The MM- and GG-blocks were further fragmented using alginate M-lyase (Lundqvist *et al.*, 2012) and alginate G-lyase (Thomas *et al.*, 2013), respectively. The enzymatic degradation and the purification of the released oligosaccharides was performed as described previously (Thomas *et al.*, 2013).

### Generation of alginate-directed monoclonal antibodies

Six rat MAbs were derived subsequent to immunization with neoglycoprotein immunogens, prepared by coupling either GG-blocks (BAM10), MM-blocks (BAM7), or alginate from DuPont (BAM6, 8, 9, 11), to BSA by activation with 1-cyano-4-dimethylaminopyridium tetrafluroborate (CDAP) (Lees *et al.*, 1996). In each case, two male Wistar rats were each injected with 250 μg of alginate–BSA conjugate in complete Freund’s adjuvant administered subcutaneously on day 0, and the same was administered with incomplete Freund’s adjuvant ~30, 60, 120, and 160 d after this. A pre-fusion boost of 100 μg of alginate–BSA in 1 ml of PBS was administered prior to spleen removal. Hybridoma production and cloning procedures were performed as described (Willats *et al.*, 1998).

### Enzyme-linked immunosorbent assays

ELISAs were performed in 96-well microtitre plates (Maxisorb, NUNC) coated with 100 μL per well of antigen (50 μg ml^–1^) in phosphate-buffered saline (PBS; 137 mM NaCl) overnight at 4 °C. Unbound antigen was washed out using tap water, and 200 μl of blocking solution of 5% milk powder in PBS (MP/PBS) were added per well. After 1 h at room temperature, plates were rinsed in tap water and 100 μl of hybridoma supernatant in MP-PBS were added at the desired dilution. Plates were incubated at room temperature for 1.5 h, washed with tap water, and then incubated with secondary antibody [rabbit anti-rat IgG, whole molecule, coupled to horseradish peroxidase (HRP), Sigma-Aldrich] at a dilution of 1:1000 for 1.5 h and at room temperature. Plates were washed in tap water, and antibody binding was detected by the addition of 150 μl per well of HRP substrate [0.1 M sodium acetate buffer, pH 6.0, 1% tetramethyl benzidine, 0.006% (v/v) H_2_O_2_] and allowed to develop for 5 min before the reaction was stopped by the addition of 30 μl of 2.5 M H_2_SO_4_. For competitive inhibition ELISAs, antigen-coated and blocked microtiter plates were incubated at the same time with the primary antibody and competitor molecules (i.e. MM- and GG-blocks of alginates and oligoalginates) in MP/PBS. After incubation for 1 h, plates were processed as above.

### Isolation of *Fucus serratus* zygotes and cultivation of the embryos

Sexually mature fronds of *F. serratus* were collected in Roscoff (France; 48.43-3.59). Receptacles were cut, rinsed, and dry stored at 4 °C in the dark for up to 2 weeks. Gametes were released by placing the receptacles in filtered seawater in daylight. Fertilization occurred within 15 min of mixing sperm with oospheres. After 1 h, the resulting zygotes were washed twice in filtered seawater and filtered through a 100 µm nylon mesh. Zygotes were held on microscope slides and allowed to grow in seawater under unidirectional light at 16 °C. A photoperiod of 12 h light and 12 h darkness was used. For the photo-polarization experiment, zygotes were cultured in filtered seawater on microscope slides and in unidirectional light (L1) beginning at 1 h after fertilization (AF). At 6 h AF, the zygotes were allowed to grow in unidirectional light (L2) oriented 180 ° from the first light source. At 24 h AF, the zygotes were observed for the direction in which rhizoid development had occurred.

In certain cases, *Fucus* zygotes were cultivated in the presence of the β-galactosyl-Yariv reagent (βGalY; Biosupplies Pty Ltd), diluted in seawater at 40 µM and made using a 2 mg ml^–1^ stock in 0.15 M NaCl. A first series of incubations started 1 h AF and continued until the time of observation. A second series of incubations was performed between 1 h and 6 h AF. At the end of the incubation period, the reagent was removed by rinsing the samples three times with filtered seawater and zygotes were returned to cultivation.

### Immunolabeling and fluorescence microscopy

Zygotes were fixed for 1 h in seawater containing 4% paraformaldehyde and 10% glycerol, and washed twice in PBS before immunolabeling. Fluorescence imaging of MAb binding to samples was achieved with indirect immunofluorescence labeling procedures with 10-fold dilutions of hybridoma supernatants followed by 100-fold dilution of anti-rat-IgG-whole molecule–fluorescein isothiocyanate (FITC; Sigma-Aldrich, F1763) as described for other rat MAbs (Marcus *et al.*, 2008). The binding of CBM3a to the zygotes was assessed by a three-stage immunolabeling technique, described previously (Hervé *et al.*, 2009). Prior to analysis, the samples were incubated with the β-linked glucan stain Calcofluor White (Fluorescent Brightner 28; Sigma-Aldrich) for 5 min in darkness, and mounted after washing in a glycerol-based anti-fade solution (Citifluor AF3; Agar Scientific). Immunofluorescence was observed with a microscope equipped with epifluorescence irradiation (BX-60; Olympus). Images were captured with an EXI Aqua camera and Volocity software (Perkin Elmer). The bright field pictures were obtained using a Qimaging camera. Micrographs of zygotes shown are representative of analyses of at least 50 (photo-polarization and βGalY experiments) and 200 (control conditions) zygotes.

## Results

### Generation of monoclonal antibodies directed towards different alginate populations

Alginates are major polysaccharides in the cell walls of brown algae. To understand the role of alginates in the context of algal cell development, a set of molecular probes covering different alginate structures is required. As a first attempt to generate MAbs to alginate structures, isolated fractions of oligomannuronates (MM-blocks; M/G ratio of 7.4) and oligoguluronates (GG-blocks; M/G ratio of 0.06) were prepared, coupled to BSA, and used as immunogens. These immunogens led to very weak immune responses although two hybridoma cell lines were isolated. Subsequent immunization with an alginate polymer (M/G ratio of 0.9) coupled to BSA led to the isolation of several further lines secreting MAbs that bound to alginates. In total six lines were selected for cloning based on initial screening for appropriate specificities and binding characteristics. The corresponding antibodies were designated BAM6–BAM11.

**Fig. 1.**
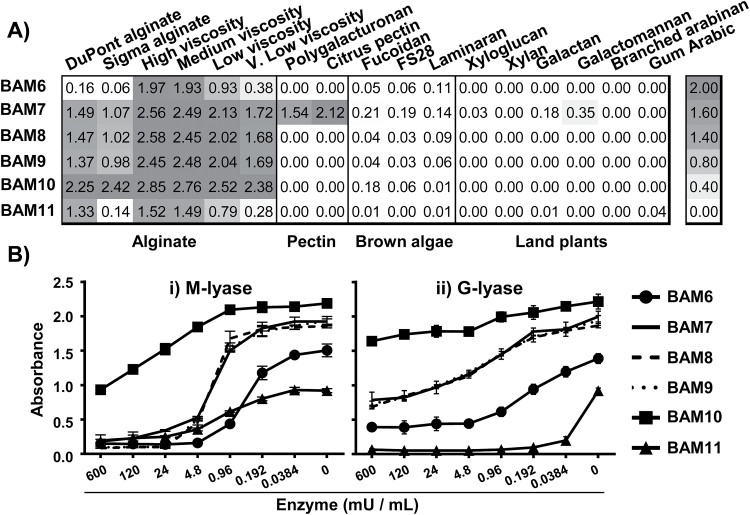
Characterization of six alginate-directed MAbs (BAM6–BAM11). (A) Heat map of epitope occurrence in a range of polysaccharides (coated at 50 µg ml^–1^) showing ELISA absorbance obtained from 25-fold dilution of MAbs. Values shown are means of four replicates and in all cases SDs were <0.1 absorbance unit. (B) ELISA analysis of loss of epitope recognition due to enzymatic cleavage of high-viscosity alginate via (i) M-lyase and (ii) G-lyase, with constant 25-fold dilution of MAbs. Note that BAM7, BAM8, and BAM9 are indistinguishable. Results are means of three replicates, and error bars represent the SD.

The alginate recognition trend for these MAbs from BAM6 to BAM11 is from a mannuronate-rich epitope for BAM6 to a guluronate-specific epitope for BAM11 as indicated by experiments outlined below. To determine the specificities of the BAM6–BAM11 antibodies toward alginates, and to assess their possible binding to other cell wall polymers, the antibodies were used to probe ELISA-based polysaccharide macroarrays in which sets of plant and brown algal cell wall polysaccharides were present (Fig. 1A). All antibodies displayed a strong binding to a range of commercially available alginate samples, and the BAM7 antibody displayed cross-reactivity with the two galacturonic-acid-rich pectin samples. No cross-reactivity towards other polysaccharides from brown algae including sulfated fucans or polysaccharides from plant cell walls was observed. Epitopes were most abundant in the high and medium viscosity alginate samples and, overall, the BAM10 epitope was the most abundant and the epitopes bound by BAM6 and BAM11 displayed the greatest differential occurrence across the alginate samples (Fig. 1A).

Analysis based on enzymatic digestion of alginate motifs was carried out in order to characterize further the alginate epitopes recognized by BAM6–BAM11. Alginate (high viscosity) was coated onto microtiter plates and treated with two specific alginate lyases prior to ELISA analysis. The alginate lyase from the marine bacterium *Pseudoalteromonas alginovora* X017 cleaves M-MM motifs (Lundqvist *et al.*, 2012) and is hereafter named as M-lyase. The alginate lyase AlyA1 from *Zobellia galactanivorans* (Thomas *et al.*, 2013) cleaves G-G motifs and is hereafter named G-lyase. The binding of BAM6 to high viscosity alginate was most susceptible to M-lyase activity, and formed a midway plateau at ~0.45 absorbance units when digested with G-lyase (Fig. 1B). This suggests that the BAM6 epitope is rich in mannuronic acid residues and may contain a restricted amount of guluronic acid residues. The BAM11 epitope is highly susceptible to the G-lyase and only moderately so to M-lyase activity, suggesting a guluronate-rich epitope. In all digestions BAM7, BAM8, and BAM9 were indistinguishable (dotted lines, Fig. 1B), suggesting that their epitopes vary little in structure. The BAM10 epitope had the highest level of detection and the least loss in response to enzymatic digestion. To explore further the relative abundance and potential differences between the alginate epitopes, cell walls from a wide range of brown algae were fractionated by a series of extractants and the solubilized materials screened by ELISA (Supplementary Fig. S1 at *JXB* online). A scatterplot analysis for all MAb combinations revealed a high correlation (average *R*^2^ of 0.980) for the occurrence of the BAM7, BAM8, and BAM9 epitopes, further supporting the observations above and suggesting similar alginate epitopes for these three MAbs—although BAM7 has a clearly different binding capacity in relation to pectic polysaccharides (Fig. 1A).

**Fig. 2.**
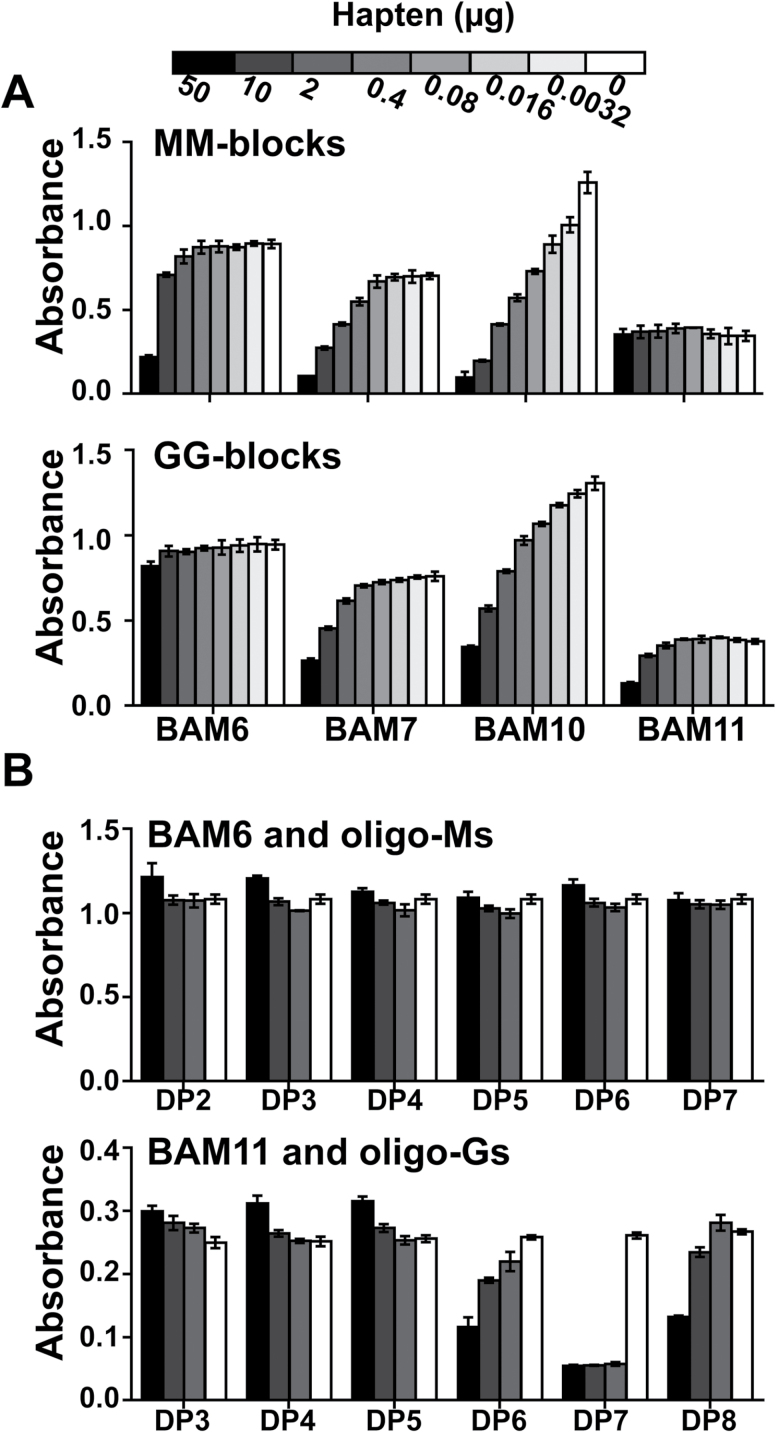
Alginate hapten inhibition ELISAs with alginate antibodies. (A) Competitive inhibition ELISAs with MM-blocks and GG-blocks of alginates with BAM6, BAM7, BAM10, and BAM11. Results are means of four replicates, and error bars represent the SD. (B) Competitive inhibition ELISAs with defined oligoalginates. BAM6 with DP2–DP7 oligomannuronates and BAM11 with defined DP3– DP8 oligoguluronates. Results are means of four replicates, and error bars represent the SD.

To determine further the structural features required for recognition, the inhibition of antibody binding to alginates was determined using competitive inhibition (hapten) ELISAs with isolated alginate MM-block and GG-block structures with a degree of polymerization (DP) ranging from 4 to ~30. BAM7, BAM8, and BAM9 displayed similar results, and only results for BAM7 are shown. BAM7 and BAM10 were most effectively inhibited by both sets of the isolated block structures, with BAM10 being most sensitive to inhibition by both MM-blocks or GG-blocks, and this is aligned with it being least susceptible to the alginate lyases. BAM6 binding was only effectively inhibited by MM-blocks, and BAM11 was only effectively inhibited by GG-blocks (Fig. 2A).

Guided by the MM- and GG-block competitive inhibition ELISAs, it was decided to attempt to determine the minimum number of M or G residues required for epitope recognition by the most specific MAbs. Digestion of the MM-blocks and GG-blocks of alginates with the M-lyase and G-lyase, respectively, was used to generate series of oligoalginates which through size exclusion chromatography were separated into individual fractions based on their DP. These procedures led to the isolation of sufficient defined DP oligosaccharides for the analysis of two MAbs. As the epitopes of BAM6 and BAM11 are potentially M and G specific, respectively, the potential of the isolated oligosaccharides to inhibit the binding of these two antibodies was determined. BAM6 was not inhibited by any of the defined oligomannuronates up to DP7 (Fig. 2B). BAM11 was most effectively inhibited by oligoguluronates with DP of 7 (Fig. 2B) which is in agreement with other indications of its binding to a guluronate-specific epitope.

In summary, BAM6 binds to a mannuronate-rich epitope but with a probable requirement for some guluronate for recognition, except GG dyads which would have been fully degraded by the G-lyase. The BAM7 antibody (and closely related BAM8 and BAM9 antibodies) most probably recognize MG regions. BAM10 recognizes a small, ubiquitous epitope of the alginate structure, most probably also MG rich, that is largely resistant to lyase action. BAM11 binds to an epitope of around seven guluronate residues.

### *In situ* detection of cell wall epitopes during early embryogenesis in *Fucus serratus*

**Fig. 3.**
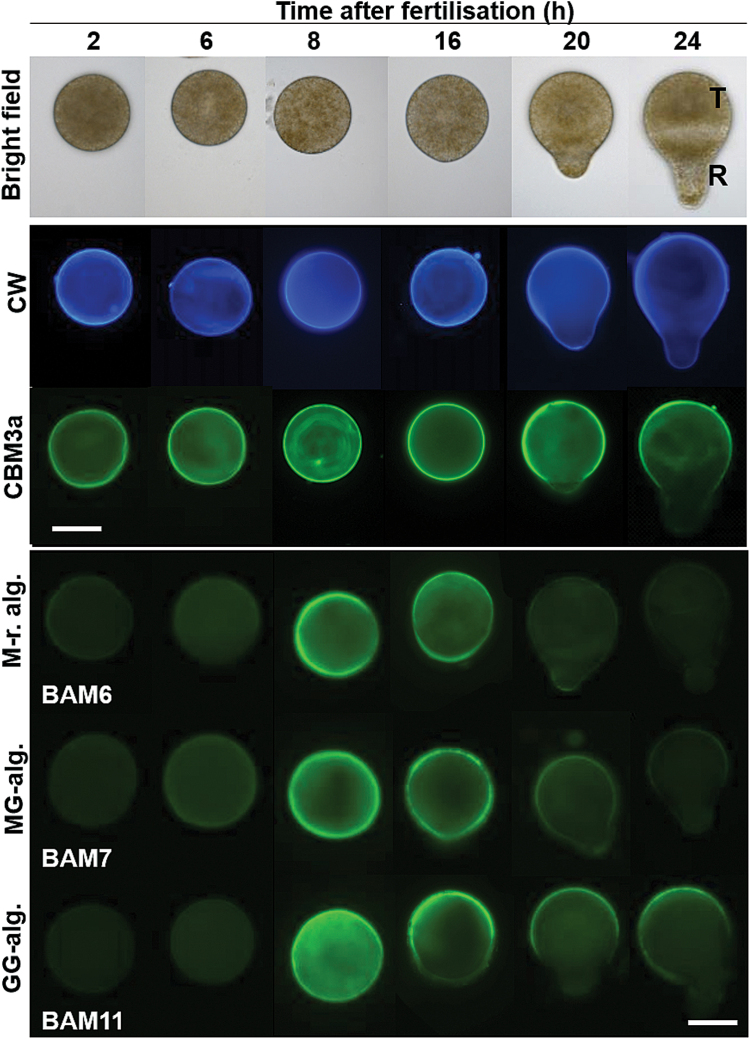
Indirect immunofluorescence labeling of cellulose and alginate populations in developing *Fucus serratus* zygotes. Bright field images on the top panel show the morphology of the zygote and early embryo at time points after fertilization. Beta-linked glucans were detected by use of Calcofluor White stain (blue fluorescence) and the carbohydrate-binding module CBM3a (green fluorescence). Detection of the BAM6, BAM7, and BAM11 alginate epitopes in equivalent individuals. T, thallus cell; R, rhizoid cell. Scale bars=50 µm.

In fucoid zygotes, the first cell division gives rise to a larger thallus cell and a smaller, polarly growing, rhizoid cell (Fig. 3). Past studies have suggested to some extent the localized secretion of cell wall structures during early embryogenesis (Quatrano *et al.*, 1991; Bisgrove and Kropf, 2001). This makes fucoid zygotes an ideal system in which to explore the capabilities of the recently produced cell wall probes while extending the previous observations on cell wall deposition dynamics. To explore the binding of both the alginate and heterofucan BAM MAbs to brown algal cell wall polysaccharides, *F. serratus* zygotes were grown in seawater and fixed at different time points AF.

#### Alginates are deposited after cellulose in zygote cell walls and are rapidly epimerized

BAM6, BAM7, and BAM11 alginate antibodies strongly bound the zygotes at moderately early stages as determined by immunofluorescence labeling, and the epitopes were strongly and uniformly distributed over the zygote wall at 8 h AF (Fig. 3). BAM8, BAM9, and BAM10 gave the same pattern of detection as BAM7 (Supplementary Fig. S2).The specificity of the developmental dynamics of the alginate epitopes was emphasized by the comparative probing of *F. serratus* zygotes at equivalent time points with both Calcofluor White stain and the carbohydrate-binding module CBM3a (Fig. 3). Both of these probes indicated that cell wall polymers were accessible at the surface of intact *F. serratus* zygotes throughout a 24 h period AF. Binding was uniformly distributed over the zygote wall at the earliest developmental stage analyzed (2 h AF), and both binding patterns became polarized to the future thallus cell when the rhizoid emerged (20 h AF). CBM3a fluorescence was barely detected at the germination zone (20 h AF) or on the rhizoid cell of two-cell embryos (24 h AF). In contrast, immunofluorescence signals for the alginate probes displayed a clear developmental dynamic and were most abundant from 8 h to 16 h AF. Some signal was observed at 6 h, albeit with much reduced intensity, and at 2 h AF alginate epitopes were not detectable. In later stages, 20 h AF onwards, the BAM6 and BAM7 fluorescence signals declined and the BAM11 epitope continued to be detected, although with decreased intensity, in a polarized manner during rhizoid emergence with a focus on the future thallus cell (Fig. 3).

#### BAM3 and BAM4 sulfated fucan epitopes are deposited in a polar manner concomitant with rhizoid emergence

**Fig. 4.**
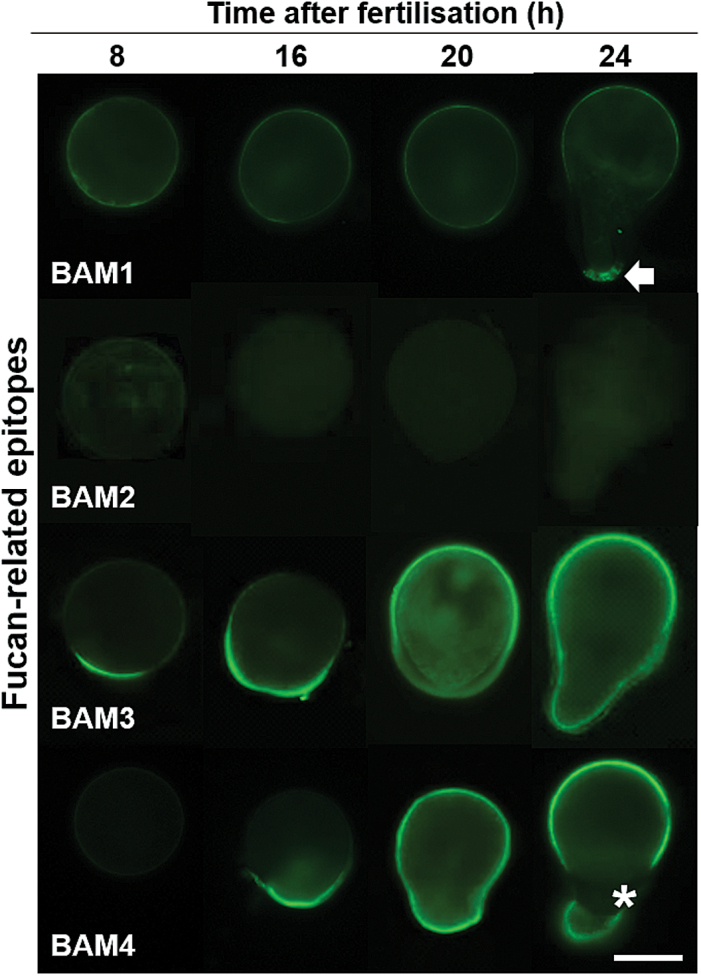
Indirect immunofluorescence labeling of fucan deposition in developing *Fucus serratus* zygotes. The zygotes/embryos were labeled at the indicated time points following fertilization. The green fluorescence shows the detection of BAM3 and BAM4 fucan epitopes in equivalent individuals. The arrow shows the presence of BAM1 epitopes at the extreme tip of the elongated rhizoid. The asterisk indicates the collar zone of the embryo. Scale bar=50 µm.

MAbs BAM1–BAM4 display strong and specific binding to preparations of sulfated fucans from cell walls of the brown algae (Torode *et al.*, 2015). BAM1 and BAM4, respectively, bind to a non-sulfated epitope and a sulfated epitope present in fucan preparations. BAM2 and BAM3 recognize additional distinct epitopes which are not yet fully characterized. These probes were used to explore the occurrence of fucan populations in relation to the zygote/embryo development. The BAM2 epitope was not detected at any stage up to 24 h AF. The BAM1 epitope was detected only at the rhizoid tip at 24 h AF (Fig. 4). In contrast, BAM3 and BAM4 bound the zygotes strongly, and the BAM3 epitope was detected at the rhizoid pole at 8 h and at the whole surface of the zygote from 20 h onwards. The BAM4 epitope displayed a similar pattern, being detected from 16 h onwards (Fig. 4). Although both BAM3 and BAM4 recognized polar depositions at early developmental stages, the BAM3 epitope consistently appeared first (8 h AF) compared with the BAM4 epitope (16 h AF). At 24 h AF, the two-cell embryo continued to grow, and there was a consistent and specific decrease in the BAM4 binding in the rhizoid collar zone (Fig. 4).

In summary, the use of the sets of cell wall probes has shown that the deposition of β-linked glucans that include cellulose occurs ahead of detectable alginate deposition and this is ahead of detected fucan deposition. There are clear contrasting dynamics to the major sets of matrix polysaccharides in that alginate occurrence gradually becomes polarized to the thallus cell and fucan deposition is initiated in a restricted location at the opposite rhizoid pole before becoming more widely distributed. The BAM3 fucan epitope is detected at the site of future rhizoid emergence at 8 h AF—ahead of visible rhizoid emergence.

### Redistribution of fucan populations associated with re-orientation of zygote growth axes

**Fig. 5.**
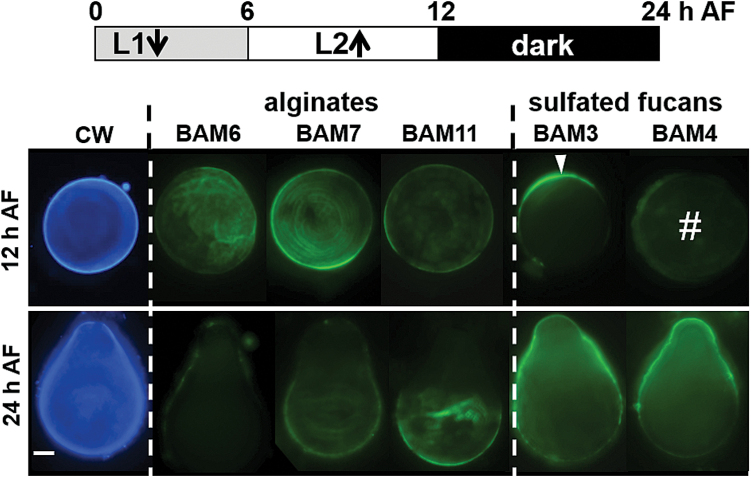
Effect of a reorientation of the growth axis on the deposition of cell wall polysaccharides in developing *F. serratus* zygotes. Zygotes were cultivated and exposed to unidirectional light (L1) while an initial growth axis was established. At 6 h after fertilization (AF), the zygotes were reoriented 180 ° from the initial light source (L2), until the time of observation. Blue fluorescence shows Calcofluor White stain. The green fluorescence shows the detection of the BAM6, BAM7, and BAM11 alginate epitopes, and also the BAM3 and BAM4 fucan epitopes. Scale bar=50 µm.

The first detectable epitope asymmetry in the zygote occurs between 8 h and 16 h with BAM3/BAM4 fucan epitopes, and during this time the axis is labile and has the potential for re-orientation by an external vector such as a unilateral gradient (Bisgrove and Kropf, 2008). We used the ability of the zygotes to photopolarize to investigate if the BAM3 and BAM4 fucan epitopes are relocated to a ‘new’ shaded hemisphere when the light gradient is reversed. Zygotes were first grown in unidirectional light (L1) during a first 6 h period AF, before being subject to a second unidirectional light (L2) gradient, orientated 180 ° to the first. At 24 h, the rhizoids emerge on the dark side of L2, indicating that all zygotes were able to reorient their L1-induced growth axis and formed a new growth axis in response to L2 (Fig. 5). At 24 h AF, when the rhizoid pole is well established, the epitope distribution patterns were similar in relation to thallus/rhizoid orientation to those observed in zygotes cultivated in a continuous L1 light direction (Figs 3–5). The zygotes were further evaluated for the location of cell wall polymers at the earlier time point of 12 h AF. As anticipated, the alginate-directed BAM6, BAM7, and BAM11 MAbs and the Calcofluor White staining showed the same uniform detection at the cell surface as observed with the controls grown in a continuous L1 light gradient (Fig. 5). However, a change in the light direction prior to axis fixation causes a re-distribution of BAM3 and BAM4 fucan epitopes. The BAM3 epitope was at the shaded side of L2 at the new future site of rhizoid germination, indicating that it had responded to change in light direction and correlated with rhizoid development. However, the BAM4 epitope was consistently not detected in a polar manner but in a weak but uniform distribution over the zygote surface (Fig. 5). These observations suggests that the set of the fucan-related polymers carrying the BAM3 epitope are part of the ASC, while those with the BAM4 sulfated epitope are a distinct population of sulfated fucans.

### Arabinogalactan-protein-binding βGal-Yariv reagent disrupts cell wall deposition and assembly during *F. serratus* early embryogenesis

**Fig. 6.**
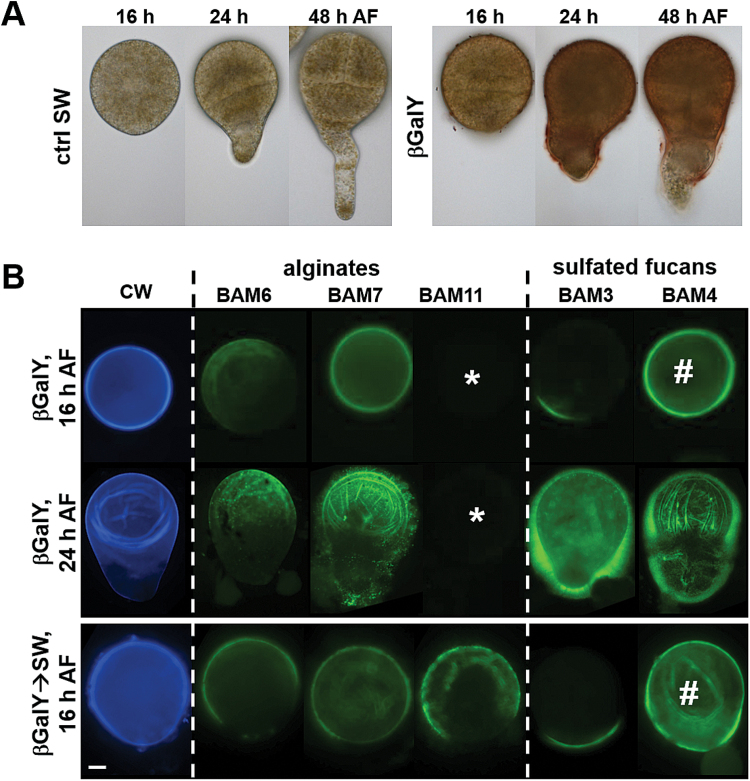
Effect of a βGalY treatment on the deposition of cell wall polysaccharides in developing *F. serratus* zygotes. (A) Bright field images showing the morphology of the treated zygotes as compared with untreated controls. (B) Indirect immunofluorescence labeling of cell wall polysaccharides. Blue fluorescence shows Calcofluor White stain. Green fluorescence shows the detection of the BAM6, BAM7, and BAM11 alginate epitopes, and the BAM3, BAM4 fucan epitopes. Zygotes were cultivated in the presence of βGalY, either from 1 h AF until the time of observation or for 8 h AF when the reagent was removed and the zygotes allowed to develop for a further 8 h in seawater (βGalY→SW). Scale bar=50 µm.

Recent work has demonstrated that *F. serratus* zygotes show an abnormal morphology when cultivated in a medium containing βGalY known to interact with AGPs (Hervé *et al.*, 2016). In the presence of βGalY, the length of the rhizoid was reduced, the collar zone expanded to give a fat rhizoid phenotype, and in extreme cases βGalY treatment results in multiple branching and abnormal cell divisions (Fig. 6A; Hervé *et al.*, 2016). To study how AGP function may be associated with specific elements of cell wall assembly dynamics, patterns of wall polysaccharide epitopes were assessed on 16-hour-old βGalY-treated zygotes, a time point where in untreated zygotes all the main classes of polysaccharides are detectable, including the fucans, which show a polar distribution at this stage. Calcofluor White staining of the βGalY-treated zygotes revealed strong fluorescence at zygote cell walls equivalent to untreated controls (Fig. 6B). At 16 h AF, the BAM6 and BAM7 alginate epitopes were uniformly detected at the surface of the treated zygotes, but to a lesser extent that in the absence of βGalY, and the detection of the guluronate BAM11 epitope was abolished entirely by the treatment (Fig. 6B). This indicates a major reduction in the metabolic development of GG-blocks in the cell walls. At 24 h AF, the BAM6 and BAM7 epitopes were more abundant in treated zygotes than in controls, further suggesting disruption of alginate dynamics, and the BAM11 epitope remained absent. In the case of the fucan-related polymers, the BAM3 epitope was detected in the βGalY-treated zygotes in a polar manner equivalent to the control. The sulfated fucan BAM4 epitope, however, was not detected in a polar manner at any stage and was uniformly distributed over the wall in the βGalY-treated zygotes at 16 h AF, although by 24 h it was detected in a pattern similar to the untreated control (Fig. 6B).

These observations indicate that AGP disruption by βGalY has impacts on subsets of both alginate and fucan polymers. Past studies have shown that the frequencies of abnormal embryos were higher with continued exposure to the Yariv reagent, compared with those observed after a short incubation period (Hervé *et al.*, 2016). This indicates that zygotes are partially able to recover from an early treatment when transferred to a fresh culture medium, suggesting that the effects on developing *F. serratus* zygotes are reversible. In order to survey if the effects of the βGalY reagent on cell wall assembly were reversible, 16-hour-old zygotes were transferred from a medium containing the reagent to fresh seawater, and assessment at 24 h showed that the epitope patterns were identical to those observed for untreated zygotes (Fig. 6B), except for the BAM4 epitope which was still uniformly distributed over the wall.

## Discussion

### New molecular tools extend capacities to study cell wall biology in brown algae

The availability of >200 MAbs targeting a variety of cell wall polysaccharides continues to be an important resource for the study of the polysaccharide-rich cell walls of terrestrial plants (Patathil *et al.*, 2012; Raimundo *et al.*, 2015). For macroalgal cell walls, the probe reservoir is limited due to less research activity, relative to plant cell walls, as well as the challenges in providing well-defined oligosaccharides for the immunization processes and/or epitope characterization. Vreeland and co-authors were pioneers in developing antibodies targeting specific algal epitopes (Vreeland *et al.*, 1984; Boyen *et al.*, 1988), but the lack of specific polysaccharides at the time limited their comprehensive characterization. The recently generated probes to fucan-related polymers (Torode *et al.*, 2015) and now BAM6–BAM11 directed to alginates, characterized herein, complement the antibodies previously obtained (Nagasato *et al.*, 2010). Additionally, the genomic information recently released from marine bacteria and brown algae allows for the characterization of recombinant enzymes involved in cell wall degradation [i.e. alginate lyases (Thomas *et al.*, 2013), fucanase (Colin *et al.*, 2006)] and alginate synthesis [i.e. GDP-mannose dehydrogenase (Tenhaken *et al.*, 2011), ManC5-E (Fischl *et al.*, 2016; Inoue *et al.*, 2016)]. Together these new implementations will be important tools for the study of cell wall biology in brown algae.

### Re-examination of cell wall assembly in the developmental model system, the *F. serratus* zygote

Biochemical and *in situ* investigations indicate that the anti-fucan BAM1–BAM4 and anti-alginate BAM6–BAM11 are highly specific to their respective polymer classes and also bind to distinct epitopes within these classes. These probes therefore permit the *in situ* tracking of relevant cell wall epitopes in developing *F. serratus* zygotes. Cellulose was detected within the first 2 h AF, ahead of alginates, which were only faintly detected at 6 h, before being abundantly present in the wall during the polar axis establishment, at 8–16 h AF. These results are in agreement with earlier observations indicating that cellulose and alginates are deposited early into the wall. However, Quatrano and Stevens (1976) were reporting the abundant detection of alginates concomitant to cellulose in zygotes from *F. vesiculosus*. This was not the case in previous studies using antibodies, which were also detecting G-blocks of alginates only at 6 h AF in zygotes from *F. distichus* (Boyen *et al.*, 1988). This discrepancy might indicate either a difference between species, or that alginates are initially incorporated into the wall in a configuration which impairs the antibody binding, or that the chemical procedure used by Quatrano and Stevens (1976), which does not differentiate between the different uronic acids, was overestimating the alginate content by incorporating cytosolic contaminants.

The three types of alginate structures, as recognized by BAM6, BAM7, and BAM11, have similar dynamics of distribution during embryo development, albeit with an earlier diminution of the M-rich epitopes relative to the guluronate-specific BAM11 epitope. This aligns with the knowledge that the distinct types of blocks are localized on the same polymer and suggest the rapid epimerization of the polymer by ManC5-Es, concomitant with alginate incorporation into cell walls. At later stages there is a localized change in alginate distribution, with a focus on the thallus cell pole, which has been poorly documented in previous literature. A disappearance of the alginate is unlikely as alginate is known to be abundantly present throughout embryo development, ranging from 40% to 60% of the total polysaccharide content (Novotny and Forman, 1974; Quatrano and Stevens, 1976). This may indicate a change in alginate structure not covered by the panel of MAbs or change in wall structure resulting in a masking of alginates. As fucan-related polymers become abundant at this stage, as indicated by the BAM3 and BAM4 epitopes, it is possible that they contribute to the masking of the alginate epitopes. Attempts to unmask alginate with a fucanase were not successful and additional fucanases would be required to explore this possibility further. Another possibility is that the MAbs bind to single alginate chains and that their binding abilities are challenged in complex composites where calcium and GG-block regions mediate self-association of the polymers. Another explanation could be that the cross-linking of alginates by phenols (Bitton *et al.*, 2006; Deniaud-Bouët *et al.*, 2014) would impair MAb accessibility to epitopes. This is consistent with the view that phenols are first incorporated at the rhizoid pole at these later stages (Schoenwaelder and Wiencke, 2000).

Previous studies have reported at least two fucan populations in *F. distichus* zygotes during early embryogenesis. A first fucan population (F1), with little detectable sulfate, and high levels of xylose relative to fucose, was detected in low amounts in walls and cytoplasm 1–6 h AF. When deposited into the wall, F1 was randomly present at the cell surface (Hogsett and Quatrano, 1975; Quatrano and Stevens, 1976). The present study reports no detection of the unsulfated BAM1 epitope, except at the extreme rhizoid tip of 24 h elongating rhizoid cells. A second population of highly sulfated fucans (F2) was first seen in the cytoplasm of the 6- to 10-hour-old zygotes but not in the cell wall until 12–14 h (Quatrano and Shaw, 1997). In the present study, the BAM3 and BAM4 sulfated epitopes were detected, in a restricted polarized manner, as early as 8–16 h AF, prior to rhizoid germination. At later stages, both these epitopes were detected across all cell walls.

The heterotrophic growth in *Fucus* zygotes is initiated by the production of a protuberance, indicative of the site of rhizoid emergence, and which further elongates through an apical growth process. Such a mechanism is reminiscent of observations made on other eukaryotic organisms having cells featuring apical growth, including land plant pollen tubes. In this system, both initiation and elongation phases of cell growth are concomitant with dynamic cell wall changes (Dardelle *et al.*, 2010; Geitmann, 2010). In the spherical *Fucus* zygote, cellulose deposition helps to organize and strengthen the wall, and the low signal of cellulose detection in the bulge wall during the initiation growth phase is congruent with a local cell wall loosening (Novotny and Forman, 1974; Bisgrove and Kropf, 2001). To date, no cellulases, expansins, or alginate lyases have been reported from genome sequences of brown algae (Cock *et al.*, 2010; Michel *et al.*, 2010; Ye *et al.*, 2015). This suggests the existence of novel mechanisms involved in the disruption of the cellulose and cell wall architecture during the transition to localized cell expansion in brown algae. In *Fucus* zygotes, sulfated components such as F2 and/or polyphenols are believed to support the strengthening of the apical wall during elongation (Schoenwaelder and Wiencke, 2000; Bisgrove and Kropf, 2001). During rhizoid cell elongation, the BAM4 sulfated fucan epitope was observed to be displaced from the collar zone, possibly indicating a region of specific cell wall modification relating to cell wall extension.

### Multifunctional roles for cell wall fucans in brown algae

Several studies on *Fucus* zygotes have demonstrated the essential role of cell walls as a source of position-dependent information required for cell polarization (Quatrano and Stevens, 1976; Kropf *et al.*, 1988; Berger *et al.*, 1994; Bisgrove and Kropf, 2001). The specific deposition of sulfated fucans (F2) at the rhizoid end during the establishment of the polar axis was indicative of a role for this polymer in the formation of an ASC bridging the cell wall, transmembrane proteins, and the cytoskeleton (Quatrano *et al.*, 1991). Both the BAM3 and BAM4 sulfated fucan epitopes have a localized distribution at the future site of rhizoid growth. However, when the polar axis fixation is challenged by an external cue such as a reversion of a light gradient, or by AGP-binding Yariv reagent, only the earlier deposited BAM3 epitope is clearly linked to the site of rhizoid initiation. These observations indicate that BAM3 and BAM4 epitopes are representative of two distinct fucan populations, with the BAM3 epitope being a positional marker for the establishment of polarity in *F. serratus* zygotes, and a likely component of the ASC, and the BAM4 epitope not being so tightly associated with axis formation.

In the brown alga *Ectocarpus subulatus*, the BAM4 sulfated epitope was modulated in relation to the external salt concentration (Torode *et al.*, 2015), which is indicative of the BAM4 epitope being involved in the osmotic adjustment at the cell surfaces through cation binding activity. In *Fucus* zygotes, cytosolic gradients of H^+^ and Ca^2+^ are generated during the establishment of polar growth (Taylor *et al.*, 1996). Increased concentration of Ca^2+^ at the rhizoid apex may play a significant role in amplifying the initial cell asymmetry. When the direction of the light vector is changed, ion gradients reposition to the new rhizoid pole (Bisgrove and Kropf, 2001). The localized insertion of sulfated fucans at the rhizoid apex is believed to participate in the regulation of the intracellular electrical field. In this study, while the BAM3 epitope was observed to be invariably initially localized at the future site of rhizoid growth, the distribution of the BAM4 fucan population undergoes a distinct dynamic change and covers the whole cell surface during reversion of the light gradient. It is possible that the population of fucans marked by the BAM4 epitope participates in the buffering of transient and local cation elevations and contributes to the redirection of the cytosolic Ca^2+^ gradient through its osmoregulation property of the cell surface.

### AGPs influence alginate modeling and cell wall assembly in the *Fucus* embryo

In *F. serratus*, AGP-binding βGalY specifically disrupts early embryogenesis, with a pronounced delay in cell elongation (Hervé *et al.*, 2016). It was postulated that βGalY was impairing cell elongation by interfering with the deposition of either cellulose or alginates. Here we show that βGalY leads to loss of detection of the BAM11 guluronate epitope at all stages of zygote development. This indicates a reduction in the *in muro* epimerization of alginates and is congruent with the knowledge that in the brown alga *Ectocarpus siliculosus*, some chimeric AGPs motifs are associated with ManC5-Es (Fischl *et al.*, 2016; Hervé *et al.*, 2016). It is therefore possible that the AGPs present in the *Fucus* embryo are chimerically associated with ManC5-Es that trigger the increase of GG-bock alginate structures. In such a system, AGPs may regulate wall rigidity through the control of the activity of appended ManC5-Es. Impairing such an AGP molecular function would therefore contribute to a softening of the wall, in particular in the collar zone, and lead in turn to the fat rhizoid phenotype, or more extreme abnormalities.

βGalY not only caused a modification in the modeling of alginates, but the BAM4 fucan epitope was also incorrectly distributed over the zygote cell surface. Instead of being polarly localized at the future germination site, the BAM4 epitope displayed a uniform binding equivalent to that observed during the redirection of the growth axis by light reversion. In land plants, AGPs have been identified as important calcium-binding molecules and proposed to act as calcium flux capacitors that regulate plant growth (Lamport and Várnai, 2013). The fucan population represented by the BAM4 epitope may be responsive to some mechanistic link between disrupted alginate structure and fucan or indicating an addition role for a distinct AGP molecule that is involved in ion fluxes and electrical gradients concomitant with polar growth, as discussed above.

In summary, with new sets of probes for alginates, described here, and fucans (Torode *et al.*, 2015), some insights have been obtained into the role of cell walls in the mechanisms underpinning zygote polarization and development in *F. serratus*. These observations emphasize the complexities of these two brown algae matrix polysaccharide classes and perhaps also the likelihood that a full coverage of polysaccharide structures by molecular probes has not yet been achieved for these polymers. Nevertheless, the isolated MAbs are useful additions to the brown algae cell wall tool box.

## Supplementary data

Supplementary data are available at *JXB* online.

Figure S1. Scatterplot matrix of BAM6–BAM11 MAb binding to sequential cell wall extracts of a range of diverse Fucale and Laminariale brown algae.

Figure S2. Indirect immunofluorescence labeling of the deposition of MG-alginates (BAM7, BAM8, BAM9, and BAM10 epitopes) in the early embryo in *F. serratus*.

Supplementary Data

## Abbreviations:

AF: after fertilization
AGP: arabinogalactan-protein
ASC: axis-stabilizing complex
BAM: brown alga monoclonal antibody
DP: degree of polymerization
M: β-d-mannuronic acid
MAb: monoclonal antibody
ManC5-E: mannuronan C5-epimerase
G: α-l-guluronic acid
βGalY: β-galactosyl-Yariv reagent.
