# Supplementary material for: Dynamics of cell wall assembly during early embryogenesis in the brown alga Fucus
Source: J Exp Bot. 2016 Oct 6;67(21):6089–100. doi: 10.1093/jxb/erw369 (PMC5100021; doi:10.1093/jxb/erw369)
Supplement: Supplementary Data [file supp_67_21_6089__index.html]

Dynamics of cell wall assembly during early embryogenesis in the brown alga Fucus — Dynamics of cell wall assembly during early embryogenesis in the brown alga Fucus — Supplementary Data 

# Dynamics of cell wall assembly during early embryogenesis in the brown alga *Fucus*

## Supplementary Data

Data files

- supplementary\_figures\_S1\_S2.pdf - Supplementary Data
