# Supplementary material for: Dynamics of cell wall assembly during early embryogenesis in the brown alga Fucus
Source: J Exp Bot. 2016 Oct 6;67(21):6089–100. doi: 10.1093/jxb/erw369 (PMC5100021; doi:10.1093/jxb/erw369)
Supplement: Supplementary Data [file supp_erw369_supplementary_figures_S1_S2.pdf]

Torode *et al*. Supplementary Figure S1

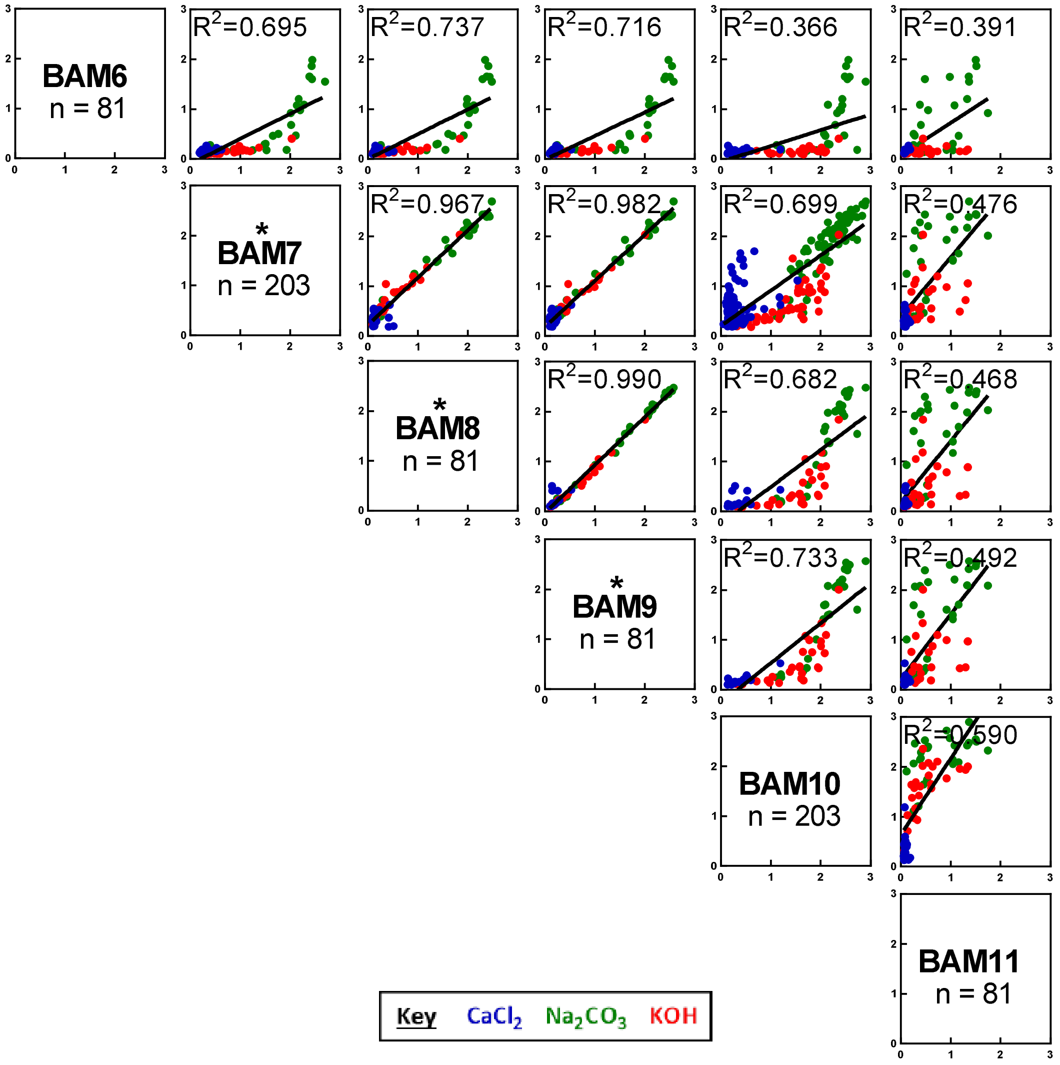

Fig. S1. Scatterplot matrix of MAb binding to cell wall extracts of brown algae. Cell wall extracts were obtained from various Fucale (*Ascophyllum nodosum, Fucus serratus, F. spiralis, F. vesiculosus, Halidrys siliquosa, Himanthalia elongata, Pelvetia canaliculata, Sargassum muticum*) and Laminariale (*Chorda filum, Laminaria digitata, L. hyperborea, L. ochroleuca, Saccharina latissima, Undaria pinnatifida*) species. Results from ELISA experiments of MAb binding to cell wall extracts of $CaCl_2$ (blue), Na_2CO_3 (green) and KOH (red) solubilized fractions were plotted as X,Y co-ordinates for each combination of the BAM6 to BAM11 MAbs. Cell wall extracts were coated at 50 µg/ml, and MAbs were used at 25-fold dilution. R^2 values are the linear line of best fit (black line) for all cell wall extracts combined. n for each scatterplot is the lowest of n of the two MAbs. * = MAbs with similar binding profiles and probable similar epitopes.

Torode *et al*. Supplementary Figure S2

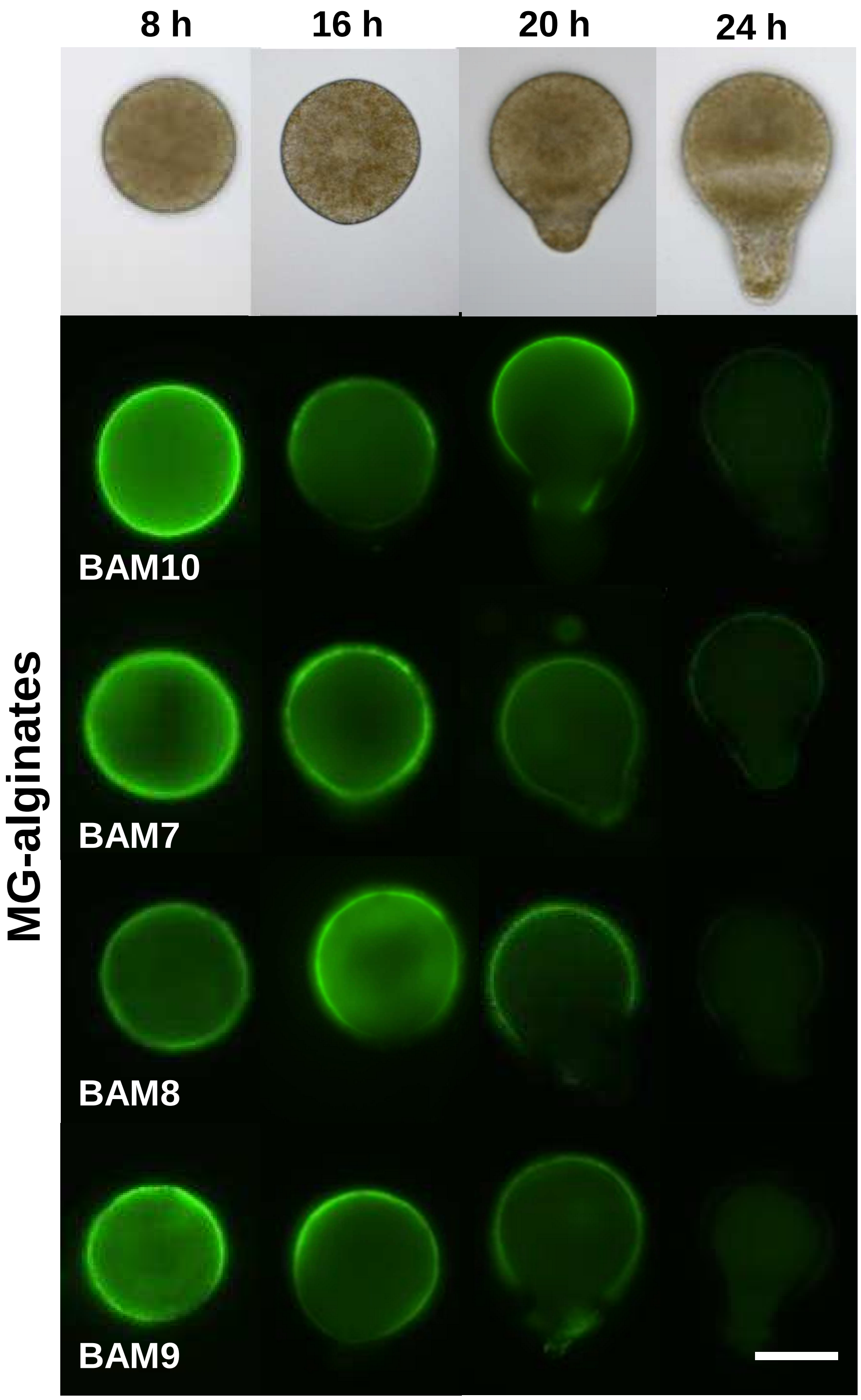

Fig. S2. Indirect immunofluorescence labelling of the deposition of MG-alginates in the early embryo in *F. serratus.* Bright field images show the morphology of the zygote and early embryo at time-points following fertilization. Green fluorescence shows the detection of the BAM7, BAM8, BAM9 and BAM10 epitopes in equivalent individuals. Scale bar = 50 μm.
